# Evaluating a sexual and reproductive health and rights programme within Swedish for Immigrants education: a study protocol

**DOI:** 10.1080/16549716.2026.2657089

**Published:** 2026-04-15

**Authors:** Tanja-Tatiana Lukin, Anna Andren, Herborg Holter, Malin Bogren

**Affiliations:** a Regionhälsan Västra Götalandsregionen, Ungdomsmottagningen Hisingen, Gothenburg, Sweden; b Department of Women’s and Children’s Health, Karolinska Institutet, Stockholm, Sweden; c Institute of Health and Care Sciences, University of Gothenburg, Gothenburg, Sweden

**Keywords:** Migrant health, health education, implementation science, sexual and reproductive health and rights, Swedish for immigrants

## Abstract

Sexual and reproductive health and rights (SRHR) are central to health equity, yet migrants in Sweden, especially from low-income countries, experience linguistic, cultural, and structural barriers to SRHR information and services. Although migrants are prioritised in Sweden’s national strategy for SRHR, no evaluation has examined SRHR education embedded within language programmes, limiting culturally responsive and scalable interventions. This protocol describes a study evaluating an SRHR programme integrated into Swedish for Immigrants (SFI), the national language programme for newly arrived adults.

The study explores implementation processes, experiences of participants and implementers, and changes in SRHR knowledge, attitudes, behaviours, and healthcare navigation. Guided by implementation research principles and the UK Medical Research Council process evaluation framework, four qualitative sub-studies will be conducted across participating SFI sites. Semi-structured interviews and ethnographic fieldwork will involve midwives, SFI teachers, municipal stakeholders, and adult migrant learners. Data will be analysed using a combination of deductive and inductive qualitative content analysis and an ethnographic – hermeneutic approach, with process evaluation examining fidelity, adaptations, mechanisms of impact, and contextual determinants.

The study is expected to identify key facilitators and barriers influencing programme delivery, acceptability, and relevance. Findings will inform evidence-based recommendations to strengthen cultural adaptation, implementation strategies, and learner engagement. Anticipated impacts include improved individual SRHR knowledge and health literacy, enhanced institutional capacity for inclusive SRHR promotion, and more equitable public service delivery at the societal level.

**Trial registration:** ISRCTN 63,513,002 (registered 27 November 2025).

## Paper Context

- **Main findings:** This study examines the implementation and anticipated impact of a culturally adapted sexual and reproductive health and rights education programme embedded within Swedish for Immigrants education.
- **Added knowledge:** The study provides new knowledge on how sexual and reproductive health and rights education can be structurally integrated into adult language education, using an ecological perspective combined with implementation science.
- **Global health impact for policy and action:** The findings may inform policymakers and practitioners about scalable and equity-oriented approaches to integrate sexual and reproductive health and rights education into adult education systems in migrant-receiving countries.

## Background

Sexual and reproductive health and rights (SRHR) are fundamental to overall health and well-being [1]. Although Sweden reports generally favourable SRHR outcomes, inequalities persist, particularly among migrants with low socioeconomic position [2]. With more than two million residents of migrant origin [3], addressing these disparities constitutes both a public health priority and a human rights obligation.

Global migration patterns have increased substantially in recent decades, driven by conflict, war, and economic instability, with over 244 million people currently living outside their country of origin [4]. During and after migration, many individuals face heightened vulnerabilities affecting their sexual and reproductive health and rights. Ensuring equitable access to SRHR information and healthcare is essential for empowering people to make informed decisions, develop critical awareness, and protect their well-being free from coercion, discrimination, and violence, recognising sexual and reproductive health and rights as fundamental human rights [5,6].

Migrants in high-income countries, including Sweden, encounter several barriers to SRHR information and healthcare services [7]. On an individual level, low health literacy, limited knowledge of healthcare systems, lack of trust in care providers, and unfamiliarity with body anatomy all restrict access [8–12]. At the structural and institutional levels, discrimination, limited cultural competence among health care professionals, language difficulties, and differing societal norms may further exacerbate inequities. These barriers collectively contribute to delayed care-seeking, preventable health complications, and persistent inequalities in sexual and reproductive health and care [13–17].

European research highlights significant unmet educational needs among migrants related to reproductive health, contraception, anatomy, communication, and cultural norms [18]. Swedish studies likewise show considerable SRHR knowledge gaps among newly arrived migrants. Addressing these requires educational approaches tailored to migrants’ linguistic, cultural, and social contexts [18,19].

Sweden’s National Strategy for SRHR identifies migrants as a priority population and emphasises the need for education-based interventions within formal and non-formal learning environments, grounded in a human right – based approach [2]. Despite this, few scientifically evaluated SRHR programmes exist for migrants in Sweden [1,2]. Evidence is urgently needed to guide effective, equitable, and rights-based interventions [1].

To understand how SRHR learning unfolds in migrants’ everyday lives, this study draws on McLeroy’s socio-ecological model, which situates health behaviour within interacting individual, interpersonal, organisational, community, and policy-level factors [20]. An ecological perspective is essential to design interventions that are culturally responsive, contextually grounded, and adaptable.

Situated within a context of migration and SRHR inequities, this study evaluates the implementation and impact of a sexual and reproductive health and rights programme structurally integrated into adult language education, with relevance for policy and practice in migrant-receiving contexts.

## Aim

The overall aim of this study is to evaluate the implementation and impact of a sexual and reproductive health and rights programme integrated into Swedish for Immigrants (SFI) education in a large urban city in Sweden. The specific aims are to:

- Identify factors influencing the implementation of the SRHR programme.
- Examine key determinants of acceptability and perceived relevance of the SRHR programme among adult migrants enrolled in SFI education.
- Explore factors that may facilitate or hinder the learning of SRHR in SFI education.
- Assess the outcomes and effectiveness of the SRHR programme within the SFI context.

### Hypothesis

We hypothesise that integrating a culturally tailored SRHR education programme into the SFI education will be feasible to implement and acceptable to both educators and migrant participants. Participation is expected to support increased SRHR knowledge and contribute to changes in participants’ attitudes, communication, and confidence in navigating healthcare services. At the organisational level, we anticipate identifying contextual and structural factors that support or challenge long-term integration, providing insights relevant for scale-up and equity-oriented public health planning.

## Methods

### Study design

**Figure 1.**
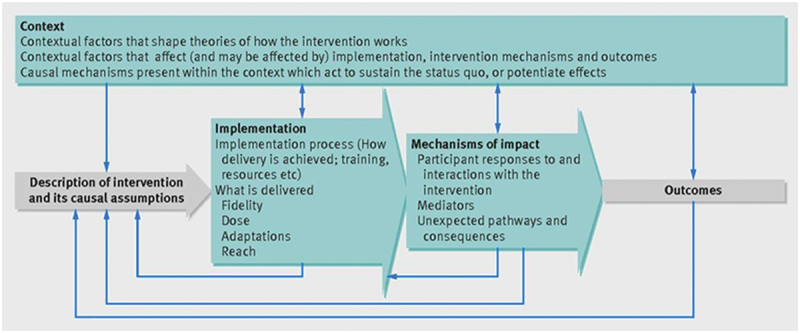
Evaluation framework by Moore et al [21].

This study draws on the implementation of science and is guided by the UK Medical Research Council (MRC) framework for process evaluation of complex interventions [21]. The framework includes detailed understanding of the intervention, contextual influences, implementation processes, mechanisms of impact, and outcomes (Figure 1).

### Study setting and intervention

Swedish for Immigrants (SFI) is a state-funded component of municipal adult education (Komvux) and is offered free of charge to individuals aged 16 and older who need to learn Swedish. In Sweden, municipalities are responsible for SFI education, while regional health services carry responsibility for health promotion, including SRHR. In addition to language instruction, SFI provides civic orientation and introduces learners to central aspects of Swedish society. Because SFI reaches migrants early in the settlement process – municipalities are mandated to offer SFI as soon as possible after arrival and within 1 month for participants in the national establishment programme – it represents a key platform for promoting awareness of sexual and reproductive health and rights [22,23].

In response to this, midwives at a midwifery clinic developed and piloted a culturally adapted SRHR education programme called ‘Sexual Health and Rights for All,’ in 2023. The programme was piloted in a district where 83% of the population is foreign-born and where socioeconomic and health disparities, including lower educational attainment and poorer health outcomes, are well documented [24,25]. The programme primarily reaches newly arrived migrants enrolled in SFI, with diverse migration trajectories, educational backgrounds, and lengths of residence in Sweden. The intervention comprises three thematic sessions of 2.5 hours each, covering SRHR, laws, individual rights, and family planning. Sessions are held separately for men and women, facilitated by two midwives to promote continuity and trust. The sessions were delivered within existing SFI classes, with group sizes typically ranging from approximately 10 to 20 participants. Depending on local organisation, the programme is either integrated into civic orientation or delivered as a stand-alone module for learners in Study Path Two (levels B–D). The programme relies on dialogue-based sessions, and a minimum level of Swedish language comprehension was therefore considered necessary to enable meaningful participation in discussions about SRHR. Participants in Study Path A may subsequently progress to higher levels within SFI and participate in the programme at a later stage. The programme aims to strengthen access to SRHR information and support participants in navigating the Swedish healthcare system, rather than to prescribe personal or cultural values.

### Data collection

#### Interviews

Data will be collected at five out of ten SFI locations where the SRHR programme has been piloted, representing the sites where the full set of education sessions has been delivered. Sites will be purposively selected to capture variation in how the programme is operationalised across SFI locations. Semi-structured interviews will be conducted with midwives, SFI staff, and municipal stakeholders, as well as with participants shortly after completing the programme and 1 year following completion. Participants who consent to follow-up interviews will be asked to provide contact details, which will be stored securely and separately from the research data. Access will be restricted to the research team and handled in accordance with applicable data protection regulations. The number of interviews will be guided by predefined target ranges for each participant group, while allowing flexibility to ensure sufficient depth and thematic saturation in the analysis. Interviewing participants at two time points allows us to capture both immediate responses and longer-term reflections on knowledge retention, application, and potential behavioural change. Interviews will explore contextual factors, barriers and facilitators, and changes in SRHR knowledge, attitudes, and use of healthcare services. Example questions included participants’ experiences of the sessions, perceived changes in SRHR knowledge, communication, and healthcare navigation, as well as midwives’ and SFI staff’s perspectives on programme delivery, barriers, and facilitators for implementation. They will be conducted in person or online, with interpreter support if needed, recorded, transcribed verbatim, and analysed using inductive content analysis in combination with theory-informed deductive perspectives drawn from the Medical Research Council implementation framework [21].

#### Observations

Focused ethnographic fieldwork will be conducted to explore how migrants engage with, interpret, and apply SRHR knowledge within the classroom setting. Participant observation, complemented by informal conversations when appropriate, will be carried out across all three sessions in four groups (two women’s and two men’s groups), with each group observed across the three programme sessions, totalling 12 observations. The groups will be purposively selected to capture variation in classroom interactions while allowing for more in-depth observation across the full set of programme sessions. Rather than using structured fidelity checklists, the observations focus on how the programme unfolds in practice by capturing classroom interactions and factors shaping how the sessions are delivered and experienced. This approach allows for a rich, contextualised understanding of classroom dynamics, interpersonal interactions, and the cultural and social factors that shape learning processes relevant to programme implementation. Data will comprise detailed field notes, reflective memos, and documentation of informal conversations, capturing both verbal and non-verbal behaviours, peer-to-peer interactions, and educator strategies. Observations will focus on teaching approaches, student engagement, questions and discussions raised, and any challenges encountered during the sessions, including structural or logistical barriers [26,27].

### Data analysis

The individual and focus group interviews will be transcribed verbatim. In the data analysis, we will apply an abductive analytic approach, combination of inductive and deductive approaches using content analysis [28]. The deductive component will be guided by the Medical Research Council framework for process evaluation, focusing on implementation, mechanisms of impact, and outcomes [21], as well as McLeroy’s socioecological model attending to individual, organisational, community, and policy-level factors shaping SRHR learning processes [20].

The inductive analysis will allow for the identification of emergent categories related to participants’ experiences of learning and change. Analysis will proceed through iterative coding, categorisation, and theme development [28]. Particular attention will be paid to participants’ narratives describing changes in knowledge and understanding, communication practices related to SRHR, and perceived ability to access and navigate healthcare services [28]. To analyse change over time, accounts of participants’ experiences will be compared within and across interviews (e.g. between earlier and later reflections, and across participant groups) to identify reported shifts in knowledge, attitudes, and behaviours. These shifts will be interpreted in relation to programme components and contextual factors, in line with the process evaluation framework [21]. Participant observation will be analysed based on field notes, reflective memos, and discussions, and follow the principles of the ethnographic iterative analysis process. This involves repeated reading of the material and movement between individual observations and the broader classroom context to identify patterns in how learning processes and behavioural expressions unfold in practice [26,27].

## Discussion

This study addresses a clear gap in evidence regarding culturally responsive, structurally embedded SRHR education for migrants, responding to international calls for equity-oriented and context-sensitive interventions [29,30]. In Sweden, where migrants face well-documented barriers to SRHR information and services [7–9], embedding SRHR learning within SFI offers a promising strategy for addressing these inequities. Because SFI reaches newly arrived migrants early in their resettlement, it provides a structurally anchored and high-reach platform for strengthening SRHR knowledge and promoting rights. Institutional or mandatory settings are commonly used for educational and public health initiatives (e.g. school-based health education) and may provide structured opportunities for access to information and dialogue [31,32].

International experiences demonstrate that integrating health literacy or SRHR content into language and civic orientation programmes can improve migrants’ navigation of health systems, decision-making, and confidence discussing sensitive topics [33–36]. Similar approaches have been described in European contexts, where SRHR initiatives for newly arrived migrants have been embedded within existing educational or health systems, highlighting both opportunities and implementation challenges [37,38]. Such findings align with the rationale for situating SRHR education within SFI, where accessibility is higher and sustainability more feasible than in short-term or stand-alone interventions, which often struggle to ensure equitable reach or long-term uptake [33,39]. Integrating SRHR education into an existing national platform such as SFI therefore has the potential to offer a stable and scalable approach. Planning for scale requires early consideration of contextual fit, organisational capacity, and adaptability, rather than treating scale-up as a post hoc activity [33,39].

Given the complexity of implementing educational interventions in multilayered systems such as adult education, the study applies both ecological and implementation science perspectives. Applying a socio-ecological perspective may help identify how individual, interpersonal, organisational, community, and policy-level factors shape engagement with SRHR information [20]. This perspective is consistent with research highlighting how migrants’ SRHR needs and engagement are shaped by intersecting social, cultural, and structural determinants across multiple levels [40]. Understanding these layers is essential for identifying mechanisms of impact and barriers that influence programme uptake. Beyond examining implementation processes, the study will also explore participants’ experiences of learning and perceived changes in SRHR knowledge, communication, and healthcare navigation following participation in the programme.

In addition to the Medical Research Council’s process evaluation framework [21], we will use two complementary implementation frameworks to deepen the analysis and discussion of how the intervention unfolds in practice: Normalisation Process Theory (NPT) [39] and the Consolidated Framework for Implementation Research (CFIR) [41]. While the Medical Research Council’s framework guides the assessment of implementation processes, mechanisms of impact, and contextual influences, NPT helps explain how the intervention may become embedded and sustained within SFI structures. NPT examines the collective and individual work required to integrate new practices, namely coherence, cognitive participation, collective action, and reflexive monitoring, offering insights into how midwives, educators, and learners engage with SRHR education in everyday routines. CFIR, used alongside NPT, enables a systematic analysis of contextual determinants, including intervention characteristics, organisational settings, individual roles, and implementation processes. Together, NPT and CFIR provide a strong foundation for understanding the interplay between context, action, and sustainability, consistent with recommendations that no single framework can fully capture the complexity of real-world implementation [39,41].

By integrating ecological theory, implementation science, and international evidence, this study is expected to generate valuable insights into how culturally responsive SRHR education can be sustainably embedded within adult education systems. Findings will contribute to global knowledge on migrant SRHR promotion and offer transferable lessons for other countries seeking to strengthen SRHR equity in line with public health goals and the 2030 Agenda for Sustainable Development [42].

Delivering SRHR education within educational settings such as SFI raises normative and ethical considerations, particularly as the programme is delivered in a mandatory educational setting while addressing sensitive topics related to voluntary healthcare. At the same time, access to SRHR is widely recognised as a human right, including the right to accurate and evidence-based information. Research suggests that healthcare professionals’ perceptions and assumptions about migrants’ SRHR-related values may shape communication and influence what information is shared in healthcare encounters [43,44]. In this context, providing structured SRHR information within institutional settings such as SFI may support more equitable access to information and dialogue, independent of individual professionals’ assumptions about what migrants need or want to know, while ensuring that engagement with SRHR information remains voluntary and free of coercion, an important consideration for implementing SRHR education within mandatory educational settings.

### Potential impact of the research

#### Policy level

Findings may inform regional and national decision-making regarding integrating SRHR into adult education. Findings can guide the development of guidelines, training strategies, and resource allocation to promote equitable SRHR education across Sweden.

#### Organisational level

The study may provide a model for sustained SRHR integration within SFI, demonstrating how municipalities, educational institutions, and regional health services can collaborate effectively. Embedding the programme within a mandatory education system ensures high reach and supports equitable access.

#### Participant level

Strengthened SRHR literacy may enhance communication, empowerment, and healthcare utilisation, while reducing stigma regarding SRHR. Improved understanding of rights and services early in the resettlement process can support long-term health outcomes, social participation, and trust in healthcare.

## Data Availability

Data sharing is not applicable to this article as no datasets were generated or analysed for this study protocol.
